# Modeling of Bentazone Leaching in Soils with Low Organic Matter Content

**DOI:** 10.3390/ijerph19127187

**Published:** 2022-06-11

**Authors:** Tadeusz Paszko, Claudio A. Spadotto

**Affiliations:** 1 Department of Chemistry, University of Life Sciences, Akademicka 13, 20-950 Lublin, Poland; 2 Embrapa Digital Agriculture, Av. André Tosello, 209, Campinas 13083-886, SP, Brazil; claudio.spadotto@embrapa.br

**Keywords:** bentazone, sorption, degradation, leaching to groundwater, soil pH, Arenosols, Luvisols

## Abstract

The aim of this study was to estimate bentazone’s potential to leach to groundwater in the Arenosols developed from sand, Luvisols developed from loamy sand or sandy loam, and Luvisols or Cambisols developed from loess, and to identify the major factors influencing bentazone’s fate in the soils. Potato and maize cultivations were simulated using the FOCUS PELMO 5.5.3 pesticide leaching model. The amount of bentazone reaching groundwater was highly sensitive to degradation parameters, water-holding capacity, evapotranspiration, organic carbon content, and pH. The highest bentazone concentrations in percolate were noted in Arenosols. The risk of bentazone concentration exceeding 0.1 μg/L was low only in Arenosols with high organic carbon content (3.0% for topsoil or higher). In Luvisols developed from loamy sand or sandy loam, the estimated bentazone concentrations in percolate were highly dependent on the climate. In Luvisols or Cambisols developed from loess, concentrations of >0.1 μg/L were the least likely due to the high water-holding capacity and high organic carbon content of these soils. The study also revealed that the FOCUS Hamburg scenario, representing the coarsest soils in the European Union with relatively low organic carbon content, does not reflect the leaching potential of Arenosols and Luvisols.

## 1. Introduction

Global pesticide use in agriculture contributes greatly to an increase in crop yields, but pesticide residues and degradation products compromise the quality of food, feed, soil, and water. In the European Union (EU), around 75% of drinking water is derived from groundwater, and its quality is continuously monitored [1,2]. According to Directive 2006/118/EC, the EU threshold value for groundwater quality standard (TGS) is set at 0.1 μg/L for a single pesticide or its metabolite, and at 0.5 μg/L for all pesticides and their metabolites detected in the monitoring procedure [1].

Plant protection products are authorized for use in the EU if they fulfil the requirements specified in Regulation 1107/2009 [3]. Among others, the approved products may not exert a harmful impact on surface water, groundwater and drinking water. The Forum for the Coordination of pesticide fate models and their Use (FOCUS) has developed procedures for assessing the leaching potential of pesticides to groundwater during the authorization procedure. Pesticide adsorption and degradation parameters are determined and used in authorized pesticide leaching programs (PEARL, PELMO, PRZM, or MACRO). The cultivation of crops treated with the examined pesticides is then simulated in nine soil- and climate-specific scenarios. In the first-tier simulations, the 80th percentiles of mean annual pesticide concentrations in percolating water at a depth of 1 m are calculated for each scenario for the last 20 years of the 26-year simulation period (*PEC_gw_*) based on the average values of *K_FOC_* (Freundlich adsorption coefficient normalized to organic carbon (*OC*) content), 1/n (Freundlich exponent), and *DT50* (half-life) for topsoils. The calculated values of *PEC_gw_* should not exceed TGS [4]. If TGS is exceeded for some scenarios or crops, higher-tier simulations are performed. The simulations rely on refined parameters (adsorption and degradation in subsoils, pH-dependent or non-equilibrium adsorption, plant uptake or pesticide volatilization, etc.), refined scenarios (adapted to the intended use in terms of soil, crops, and climate), or both [4].

Bentazone (3-isopropyl-1*H*-2,1,3-benzothiadiazin-4(3*H*)-one 2,2-dioxide) is widely used as a post-emergence herbicide around the world. The compound is applied mainly to control broadleaf weeds and sedges [5]. Bentazone is a weak organic acid with *pK_a_* = 2.86 [6,7], and in soils with pH > 5, it exists almost exclusively in anionic form, and therefore, it is weakly sorbed. In most studies, *K_FOC_* values were determined within the range specified in the EU dossier from 2015, i.e., 3.0 to 175.6 μg^1^^−1/n^ (mL)^1/n^ g^−1^ [8]. Bentazone is adsorbed mostly on soil organic matter, and at low pH also on quartz. The adsorption of bentazone in soils can be well described with the pH-dependent adsorption model, which assumes that the anionic form of herbicide is adsorbed on organic matter and the neutral form is adsorbed on organic matter and sand [7]. This compound is relatively quickly degraded in topsoils. In the cited EU dossier, *DT50* values for topsoils were in the range of 8.2–35.0 d in the laboratory and 3.0–31.3 d in the field studies. 

Monitoring studies have shown that bentazone was rarely detected or was detected at very low concentrations in topsoil samples [9,10]. However, this compound was frequently detected in surface water samples (3–69%) at maximum concentrations from various studies of 0.04–0.701 μg/L [10,11,12,13,14]. Bentazone was less frequently detected in groundwater (14–32%), but its maximum concentrations were higher (0.29–10.6 μg/L) [15,16,17]. The concentrations of major bentazone metabolites (N-methyl-bentazone and 2-amino-N-isopropylbenzamide) were below TGS [18,19,20]. As bentazone can reach groundwater, it has often been used as a model herbicide in field, lysimetric, and modeling studies [19,21,22].

As regards soil characteristics, the FOCUS Hamburg scenario can represent the coarsest soils of central Europe, i.e., Arenosols (AR) and Luvisols (LV). However, according to FOCUS some Polish regions are not represented by the Hamburg scenario, this is mostly because the prevailing soil types are coarser or have lower organic matter content [4]. However, possible differences in leaching potential between the Hamburg scenario and soil- and climate-specific scenarios for Poland have not been compared to date. Moreover, there are no studies assessing the effect of pH-dependent adsorption of bentazone on its leaching to groundwater.

Therefore, we hypothesized that TGS for bentazone may be exceeded in some soil groups of Poland. The specific objectives of the study were to: (i) identify the factors that significantly influence bentazone leaching to groundwater in the coarsest mineral soils, with particular emphasis on pH-dependent adsorption, (ii) assess the most likely ranges of *PEC_gw_* in soils, and (iii) determine the extent to which the FOCUS Hamburg scenario reflects the leaching potential of the examined soil groups.

## 2. Materials and Methods

### 2.1. Soils

Twenty-seven soil profiles (code numbers 45 to 913) were selected from the database and soil collection of the Institute of Agrophysics of the Polish Academy of Sciences in Lublin [23], and three profiles (denoted as HT, WO and SO) were selected based on the information provided by the Institute of Soil Sciences and Environmental Management of the University of Life Sciences in Lublin. Eleven profiles classified as AR [24] represent 27% of the coarsest Polish arable soils developed from sand. Eleven profiles classified as LV represent 24.7% of the soils developed from loamy sand or sandy loam, while eight profiles classified as Luvisols or Cambisols (LV&CM) represent 6.9% of the soils developed from loess or loess-like formations. The locations of the examined soil profiles are presented in Figure S1 in the Supplementary Materials. Soil properties (sand (*Sand*), silt (*Silt*), clay (*Clay*), and *OC* content (%), soil pH, water content at field capacity (*FC*) (L/L), and saturated hydraulic conductivity (*Ks*) (cm/day)) and methods of soil analysis are presented in Table S1 in the Supplementary Materials. Other soil properties are described elsewhere [7,25].

### 2.2. Adsorption Isotherms

Aqueous solutions of bentazone with a concentration of 1.5, 3.0, 4.5, 6.0, and 7.5 mg/L were prepared using a certified analytical standard (99.8 ± 0.1% purity; Institute of Organic Industrial Chemistry, Warsaw, Poland) and sterile redistilled water. The remaining solvents and reagents were of analytical grade or high-performance liquid chromatography (HPLC) grade.

Batch adsorption experiments were performed according to the OECD Guideline 106 [26] at a temperature of 22 ± 1 °C, with a soil–solution ratio of 1:1. Kinetic experiments [7] demonstrated that an adsorption equilibrium could be obtained within 24 h. Adsorption isotherms were determined for samples from three depths of six soil profiles with differentiated soil texture, pH, and *OC* contents: 611 (AR developed from sand; *OC* 0.03–0.68%, pH 4.1–4.6), 590 (LV developed from loamy sand; *OC* 0.08–0.93%, pH 4.7–5.0), SO (LV developed from sandy loam; *OC* 0.10–0.80%, pH 6.2–6.7), WO (LV developed from sandy loam; *OC* 0.12–0.75%, pH 4.4–5.4), HT (LV developed from loess; *OC* 0.07–0.76%, pH 4.7–6.1), and 564 (CM developed from loess; *OC* 0.49–1.23%, pH 6.2–6.6). Briefly, soil samples with a dry weight of 2 g each were placed in 10 mL glass tubes, 1.735 mL of 0.012 M CaCl_2_ containing 3 × 10^–5^ M HgCl_2_ (used as a biocide) was added, and the tubes were equilibrated overnight. Next, 0.265 mL of bentazone solutions at five concentrations ranging from 1.5 to 7.5 mg/L was added to triplicate soil samples; the tubes were agitated for 24 h, centrifuged (10 min, 3300 g, 20 °C), and the aqueous phase was collected for HPLC analyses. The amount of adsorbed bentazone was calculated as the difference between its initial and final concentration in the solution. The HPLC analysis has been described in detail previously [7].

### 2.3. Modeling of Bentazone Leaching

Bentazone leaching was simulated using the FOCUS PELMO 5.5.3 one-dimensional pesticide leaching model, which estimates the vertical transport of pesticides in the unsaturated soil system within and below the plant root zone. Parametrization of PELMO described below is consistent with guidelines and recommendations of FOCUS for simulations required for registration of pesticides in the EU [4,27]. The cultivation of maize and potatoes was simulated because in Poland; these crops occupy the largest area (11.6% and 2.8%, respectively, in 2019 [28]) of all crops where bentazone is used for plant protection. It was assumed that plant emergence, maturity, senescence, and harvest dates were identical to those in the FOCUS Hamburg scenario. A single bentazone dose of 0.960 kg/ha was applied 12 days after emergence in maize (BBCH 14) and 5 days after emergence in potatoes (BBCH 12). The doses were adjusted for crop interception (calculated with AppDate 3.06 software [27]), and the modeled values were 0.720 kg/ha for maize and 0.816 kg/ha for potatoes.

Bentazone characteristics, including molecular mass (240.3 g) and Henry’s law constant at 25 °C (7.2 × 10^–5^ Pa m^3^/mol), were obtained from the Pesticide Properties Database [20]. The *DT50* values obtained in our previous study [25] for bentazone degradation in HT, WO, and SO profiles at 25 °C and 40% water-holding capacity were recalculated to 20 °C using the Arrhenius equation, and to water content at *FC* (10 kPa; estimated using pedotransfer functions of PELMO) using the Walker equation [4]. The resulting *DT50* values for the Ap horizon of HT, WO, and SO profiles were 4.3, 3.4, and 16.6 d, respectively (geometric mean-6.2 d), and they were within the range of values specified in the EU dossier (3.0–31.3 d; geometric mean-7.5 d, n = 6) [8]. The geometric mean values of 7.5 d and 6.2 d were used in simulations as the most reliable values. 

The default FOCUS values of 1:0.50:0.30 were tested as the depth-dependent degradation factors (DDF-ratio of *DT50* values for topsoil and the respective subsoil horizon). The DDF values of 1:0.42:0.11 that were previously determined for three pesticides, including bentazone [25], were also used. The applied temperature correction factor for degradation (Q10 = 2.58), Walker moisture exponent (0.7), and plant root uptake factor (0.5) are default values or the values recommended by FOCUS [4]. The thickness of the topsoil and two subsoils was set at 25 cm, 35 cm, and 40 cm, respectively. The dispersion length of 100 cm profile layers was set at 5 cm [4]. The macropore flow option was not used during simulations because in coarse soils, including AR and LV, pesticides are transported to groundwater primarily via piston flow [18]. 

Climate data for 1990–2009 were obtained from the Hydro-Meteorological Station in Lublin-Radawiec (51°13′ N, 22°24′ E; hereinafter referred as the Lublin climate) of the Institute of Meteorology and Water Management. The 26-year simulation period was obtained by repeating the first 6 years. Hamburg climate files were also used.

To ensure that program was correctly parametrized, the input parameters used in the EU dossier [8] were tested: DDF-1:0.5:0.3, *DT50* = 7.5 d, *K_FOC_* = 30.2 μg^1^^−1/n^ (mL)^1/n^ g^−1^, and 1/n = 0.97. For the above parameters, the values of *PEC_gw_* for the Hamburg scenario, calculated based on the default *FC* and *WP* (water content at wilting point; 1600 kPa), and the *ET* (daily potential evapotranspiration) values from the climate files, were identical to the PELMO values published by EFSA [8] at 0.020 μg/L for both crops. If potential evapotranspiration and water content at *FC* and *WP* are unknown, the Hamon equation and PELMO pedotransfer functions, created based on the results of lysimetric studies carried out during program validation, are recommended options, respectively [27]. When the above settings were applied to the Hamburg scenario, *PEC_gw_* was determined at 0.020 μg/L for maize and 0.017 μg/L for potatoes. Thus, it was assumed that both settings were sufficiently accurate to be used as default settings in simulations of Polish soil profiles.

*K_F_* and 1/n values determined in batch experiments (Table S2 in the Supplementary Materials) were used in the simulations of six profiles. The *K_d_* values derived experimentally by Paszko et al. [7] were used as *K_F_* values for the remaining 24 profiles, and a similar approach had been previously used by Paszko [29] and Paszko [30]. The value of 1/n, was set at 0.95 for 24 profiles based on the average experimental values in Table S2 in the Supplementary Materials. For selected simulations, *K_d_* values were predicted based on bentazone *pK_a_* = 2.86, soil pH, *OC*, and *Sand* contents with the use of the equation described by Paszko et al. [7]:

(1) $$K_{d}=\frac{0.226\;OC+0.018\;Sand}{1+10^{pH-pKa}}+\frac{0.072\;OC}{1+10^{pKa-pH}}$$

The first term of Equation (1) describes the adsorption of the neutral form of bentazone and the second term the adsorption of its anionic form. The equation was parametrized for bentazone adsorption in Polish soils, but it was characterized by a good fit to the majority of *K_d_* values in the literature (see Paszko et al. [7]).

Data were processed statistically in Statistica 13 (TIBCO Software Inc., Palo Alto, CA, USA).

## 3. Results and Discussion

### 3.1. Adsorption Isotherms

Freundlich isotherm *K_F_* values were determined in the range of 0.054–0.141 and 0.07–0.042 μg^1^^−1/n^ (mL)^1/n^ g^−1^ for topsoils and subsoils, respectively (Table S2 and Figure S3 in the Supplementary Materials). The upper threshold of *K_F_* values presented in the EU dossier (0.02–3.06 μg^1^^−1/n^ (mL)^1/n^ g^−1^) EFSA [8] is higher, which can be attributed to a much higher *OC* content in the soils (up to 3.5%; up to 1.23% in this study). The obtained *K_FOC_* values (4.4–140.0 μg^1^^−1/n^ (mL)^1/n^ g^−1^) were in the range specified in the EU dossier (3.0–175.6 μg^1^^−1/n^ (mL)^1/n^ g^−1^). Similarly to our previous study [7], *K_FOC_* values were lower in topsoils (4.4–20.7 μg^1^^−1/n^ (mL)^1/n^ g^−1^) and higher in subsoils (5.6–140.0 μg^1^^−1/n^ (mL)^1/n^ g^−1^. In the group of the tested subsoils, *K_FOC_* values were highest in AR, which indicated that quartz contributed to adsorption [7]. This result also indicates that *K_FOC_* values should not be used to compute *PEC_gw_*, because bentazone leaching will be underestimated in topsoils and overestimated in subsoils.

The obtained 1/n values (0.87–0.95 and 0.88–1.05 for top- and subsoils) were in the range indicated in the EU dossier (0.66–1.13). Low *K_F_* values for topsoils, very low *K_F_* values for subsoils and 1/n values of around 0.95 (mean value for topsoils and subsoils) indicated that bentazone is mobile in topsoils and very mobile in subsoils.

### 3.2. Influence of Degradation Parameters on Bentazone Leaching

**Figure 1:**
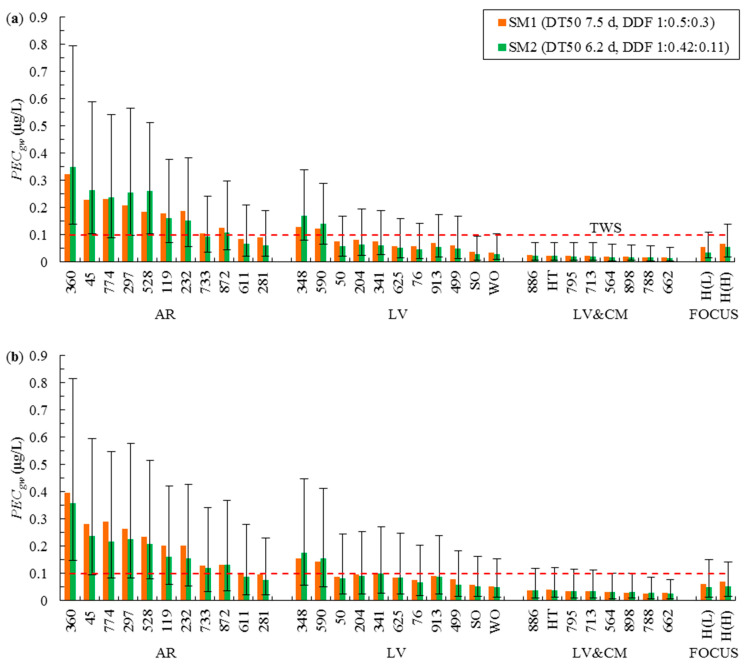
Values of *PEC_gw_* predicted during the simulations of: (**a**) Potato cultivation; (**b**) Maize cultivation. For SM2, the top and bottom of the error bars denote simulations involving *DT50* 7.5 d and 5.1 d, respectively, and DDF 1:0.42:0.11. H(L)—data for Hamburg profile and Lublin climate, H(H)—data for Hamburg profile and climate.

The degradation parameters used previously in the EU dossier simulations [8], i.e., *DT50* = 7.5 d and DDF 1:0.50:0.30, were applied in the first simulation set (SM1). The simulations were carried out in 30 profiles of Polish arable soils with *OC* content typical of the examined soil groups. In potato simulations (Figure 1), the obtained *PEC_gw_* exceeded TGS in nine out of eleven AR profiles and in two out of eleven LV profiles. In maize simulations, *PEC_gw_* exceeded TGS for ten AR and two LV profiles. In the LV&CM profiles for both crops, the computed *PEC_gw_* were below TGS. Thus, simulations revealed that TGS can be exceeded when bentazone is applied in the AR soil group.

In the second simulation set (SM2), the value of *DT50* was set at 6.2 d based on the geometric mean of *DT50* values that were derived experimentally for Polish soils. The applied value was also the median value reported in the field studies by the EU dossier [8]. The experimental DDF values of 1:0.42:0.11 for Polish soils [25] were also applied. Our previous studies [25,29,30] demonstrated that DDF values for Polish soils, especially AR and LV profiles, are lower than default FOCUS values. The DDF values for bentazone in subsoil samples from a depth of 40–50, 60–70, and 70–80 cm, were calculated at 0.31, 0.18, and 0.11, respectively, based on the work of Rodríguez Cruz et al. [31]. The results of SM2 (Figure 1) were similar to SM1 results; the estimated *PEC_gw_* exceeded TGS in eight AR and two LV profiles for potatoes, and in nine AR and two LV profiles for maize.

The simulations presented in Figure 1 indicate that values of *PEC_gw_* were affected by the properties of the examined soil groups. Significant correlations between bentazone concentrations in groundwater and soil type were previously reported by Tiktak et al. [32] and McManus et al. [14]. A monitoring study performed in the field in Vredepeel, the Netherlands [22,33] revealed that significant amounts of bentazone are leached to depths greater than 1 m in a soil developed from sand. However, in a monitoring study conducted on Luvisols and Chernozems developed from loess in the Querne/Weida catchment in central Germany [10], bentazone concentrations in surface water were determined in the range of 0.01–0.04 μg/L only, and were highly similar to the values noted in the present study in the LV&CM soil group (Figure 1).

Values of *PEC_gw_* were similar in SM1 and SM2 because the lower *DT50* value for topsoils in SM2 was compensated by lower DDF values for subsoils. Two additional simulations were carried out to determine the actual influence of degradation parameters on *PEC_gw_*. DDF values of 1:0.42:0.11 and *DT50* values of 7.5 d or 5.1 d (~65th and ~35th percentiles of the EU dossier values, respectively) were used in the simulations. The results are presented in Figure 1 as the top (*DT50* 7.5 d) and bottom (*DT50* 5.1 d) error bars in SM2. Thus, the error bars reflect ~30% variance in degradation rates. As shown in Figure 1, a decrease in the degradation rate to *DT50* 7.5 d in the arable layer led to a high increase in *PEC_gw_* in excess of TGS in all AR profiles and in most LV profiles. An increase was also observed in LV&CM profiles, but it was below TGS in most cases. In turn, when the degradation rate increased to *DT50* = 5.1 d, *PEC_gw_* in all LV profiles and in most AR profiles did not exceed TGS. Despite the above, the values of *PEC_gw_* calculated for AR were still high (median value for both crops—0.066 μg/L). 

The values of *PEC_gw_* for bentazone were also highly sensitive to changes in the degradation rate in subsoils. The differences between values of *PEC_gw_* for SM1 and SM2 at *DT50* = 7.5 d (top of the error bars in Figure 1) result from the replacement of DDF 1:0.5:0.30 with DDF 1:0.42:0.11. The increase in *PEC_gw_* was similar to that observed when *DT50* = 6.2 d was replaced by *DT50* = 7.5 d. The highest increase occurred in AR and the lowest increase was noted in the LV&CM soil group. 

The effect of degradation parameters settings on *PEC_gw_* was recently examined by Lammoglia et al. [21] in soil with the texture of clay loam (0–30 cm) to silty clay loam (90–120 cm). They found that *DT50* for topsoils was the fourth most important determinant of bentazone leaching. The results presented in Figure 1 indicate that degradation parameters exert a considerable effect on *PEC_gw_* in coarse soils. The simulations also revealed that correct degradation parameter settings for bentazone are key for obtaining reliable values of *PEC_gw_*. Despite a smaller number of topsoil observations (n = 3 vs. n = 6 in the EU dossier), the degradation parameters obtained for Polish soils gave results comparable to the simulations presented in the EU dossier. Therefore, it was assumed that these parameters (*DT50* 6.2 d and DDF 1:0.42:0.11) could be applied in further simulations.

### 3.3. Comparison of PEC_gw_ for AR, LV, and LV&CM with the Values for the Hamburg Scenario

The SM1 and SM2 simulations in the FOCUS Hamburg profile were carried out for the Hamburg climate and the Lublin climate (Figure 1). *ET* values from the climate files and default *FC* and *WP* values were used in Hamburg climate simulations, whereas the Hamon model and PELMO pedotransfer functions were used in Lublin climate simulations. Similarly to the simulations published in the EU dossier [8], values of *PEC_gw_* in Hamburg climate simulations were considerably below TGS for both crops. Values of *PEC_gw_* in Lublin climate simulations were somewhat below the Hamburg values, and the difference was attributed to lower precipitation in the Lublin climate. The temperatures in the Hamburg scenario are somewhat higher than in Lublin, whereas precipitation is much higher than in Lublin (Figure S4 in the Supplementary Materials). Since values of *PEC_gw_* were much higher for AR, the Hamburg scenario does not reflect the leaching potential of typical AR profiles. In most LV profiles, values of *PEC_gw_* were also higher than in the Hamburg scenario. Thus, the Hamburg scenario is not representative of the LV soil group either (excluding soils with the highest *OC* and the lowest pH). In turn, bentazone leaching to groundwater was higher in the Hamburg scenario than in the LV&CM soil group. 

The data presented in Figure 1 suggest that the differences in *PEC_gw_* between the Hamburg scenario and the scenarios for AR and LV profiles were related to differences in soil properties rather than to differences in climate. As suggested by FOCUS [4], one of these properties was the much higher *OC* content in the Hamburg profile (1.5% in the 0–30 cm layer and very high 1.0% content in the 30–60 cm layer) than in typical AR or LV profiles (Figure S2 in the Supplementary Materials). High *OC* content was responsible for higher bentazone adsorption and retention in the Hamburg profile. In addition, hydraulic parameters in the Hamburg scenario were not highly representative of AR because the Hamburg profile consisted of sandy loam at a depth of 0–60 cm and sand only at depths greater than 60 cm (Table S3 in the Supplementary Materials) [4].

### 3.4. Soil Properties Affecting Bentazone Leaching

Pesticide leaching to groundwater is determined by numerous processes (solute transport, degradation, adsorption, plant-uptake, volatilization), climate (temperature and precipitation), and agricultural practices (pesticide application dose and date) [21,22]. The influence of degradation parameters is discussed in Section 3.2. In the case of adsorption, values of *PEC_gw_* increase with a decrease in *K_F_* and an increase in 1/n. In this study, the effect of 1/n, pesticide dose and application date on *PEC_gw_* was not examined. In the case of *K_F_*, the means of the used *K_d_* and *K_F_* values for topsoils were 0.152^a^, 0.100^b^, and 0.081^b^ mL/g for AR, LV, and LV&CM soil groups, respectively (significant differences (*p* = 0.05) are marked with different letters in the superscripts; LSD test in one-way ANOVA), and the differences were not significant in subsoils. For details, see Table S4 and Figure S7 in the Supplementary Materials. Thus, bentazone leaching in the AR soil group should be lowest, not highest, when only *K_d_* and *K_F_* values are taken into account.

**Figure 2:**
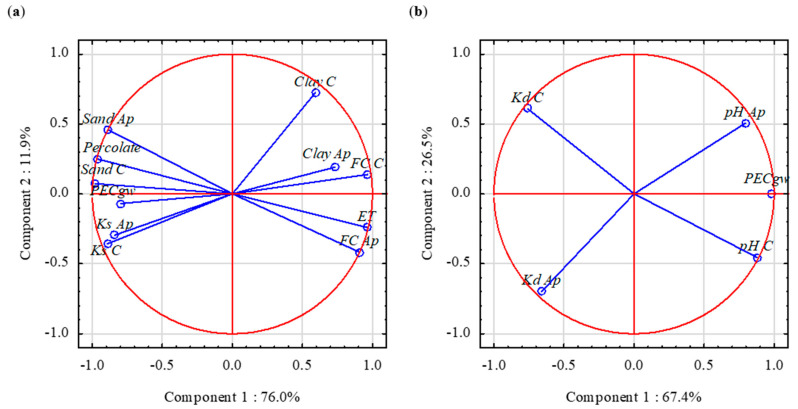
PCA results: (**a**) The effect of soil properties on *PEC_gw_* in the AR, LV, and LV&CM soil groups in SM2 simulations of potato cultivation; (**b**) The effect of soil properties on *PEC_gw_* in the AR soil group in SM2 simulations of potato cultivation.

Soil properties responsible for the highest bentazone leaching in AR, lower leaching in LV, and the lowest leaching in LV&CM (Figure 1) were determined by PCA, supported with one-way ANOVA or (when variances were not equal) by a Kruskal–Wallis test. Soil properties from the upper subsoil horizon were disregarded to obtain legible two-dimensional graphs with eigenvalues for the third component <1. PCA the SM2 of potatoes (Figure 2a) and maize (Figure S6 in the Supplementary Materials) revealed that *PEC_gw_* was strongly positively correlated with *Sand*, *Percolate* (mean annual volume of water percolating to a depth of 1 m), and *Ks*. Strong negative correlations were observed between *PEC_gw_* vs. *Clay*, *ET*, and *FC*. *Percolate* (expressed as a percentage of precipitation) was significantly higher for AR than for LV and LV&CM soil groups (27.8%^a^ vs. 25.7%^b^ and 22.8%^b^ for potatoes; 24.8%^a^ vs. 22.6%^b^ and 20.7%^b^ for maize, respectively; letters in superscript—Kruskal–Wallis test (*p* = 0.05)). Other details concerning relationships between *PEC_gw_* and *Ks*, and between *PEC_gw_* and *FC*, are presented in Figure S8 in the Supplementary Materials. Bentazone leaching to groundwater was strongly correlated with water-holding capacity in a study by Tiktak et al. [32], and with boundary hydraulic conductivity in the work by Lammoglia et al. [21].

*Percolate* was influenced not only by *FC*, *Sand*, and *Clay*, but also by *ET*, which was significantly lower for AR than for LV and LV&CM soil groups (71.9%^a^ vs. 74.1%^b^ and 76.9%^b^ for potatoes; 74.9%^a^ vs. 77.1%^b^ and 78.9%^b^ for maize, respectively; Kruskal–Wallis test (*p* = 0.05)). Thus, the lowest water capacity in the AR soil group (the mean *FC* for AR, LV and LV&CM was 0.229^a^, 0.270^b^ and 0.380^b^ mL/mL for the Ap horizon; and 0.210^a^, 0.342^b^ and 0.386^b^ mL/mL for the C horizon, respectively; Kruskal–Wallis test (*p* = 0.05)) decreased evapotranspiration and increased the volume of *Percolate*. High *Sand* content of AR and low water capacity were associated with significantly higher *Ks* values in AR than in LV and LV&CM (mean *Ks* values were 247^a^, 52^b^, and 41^b^ cm/day for the Ap horizon; and 482^a^, 6^b^ and 18^b^ cm/day for the C horizon, respectively; Kruskal–Wallis test (*p* = 0.05)), which also contributed to an increase in bentazone leaching in AR. 

Low water capacity and high water conductivity in AR group also contributed to lower bentazone volatilization from the soil surface. Bentazone volatilization (expressed in percentage of the applied dose) in AR, LV, and LV&CM was determined at 3.3%^a^, 4.2%^b^, and 4.3%^b^ for potatoes, and at 3.7%^a^, 4.5%^b^, and 4.6%^b^ for maize, respectively (LSD test in one-way ANOVA (*p* = 0.05)). However, the low Spearman’s correlation coefficients between the amounts of bentazone transported with percolating water below 1 m depth and the amount of volatilized bentazone (−0.329 for potatoes and −0.322 for maize) and high *p*-values (0.076 and 0.083) indicates that volatilization exerted a minor influence on the amount of leached bentazone. The rate of pesticide volatilization from the soil surface is correlated with evaporating water, and this process is taken into account in FOCUS PELMO [34,35].

The applied values of *K_F_* and *K_d_* did not exert a major effect on *PEC_gw_* when statistical analyses were performed for all three soil groups. However, in individual soil groups, bentazone leaching was considerably affected by adsorption, which is determined by *OC* and *Sand* contents (Equation (1)) and is inversely related to soil pH. In individual soil groups, *PEC_gw_* was negatively correlated with *K_d_*, and positively correlated with pH (Figure 2b and Figure S6b in the Supplementary Materials). In AR profiles (Figure 1), the highest values of *PEC_gw_* (profiles 360, 45, and 774) were noted when pH was in the upper range, whereas the lowest values of *PEC_gw_* (profiles 281, 611, and 733) were noted when pH was in the lower range (Table S1 in the Supplementary Materials). Surprisingly, differences in *OC* content were less significant than differences in pH. High values of *PEC_gw_* in LV profiles 348 and 590 also resulted from low sorption caused by high soil pH and low *OC* content.

### 3.5. Risk of Excessive Bentazone Leaching in AR and LV

The results of SM1 and SM2 (Figure 1) indicate that bentazone leaching to groundwater in AR and LV can exceed TGS. However, the risk of excessive bentazone leaching in AR and LV was difficult to assess from the data presented in Figure 1 or soil characteristics (e.g., range of *OC* content and pH range) for which this risk is high. The reason for the above was that profiles with typical *OC* contents were selected, whereas profiles with very high and very low *OC* contents were avoided in the examined soil groups (Figure S2 in the Supplementary Materials). 

**Figure 3:**
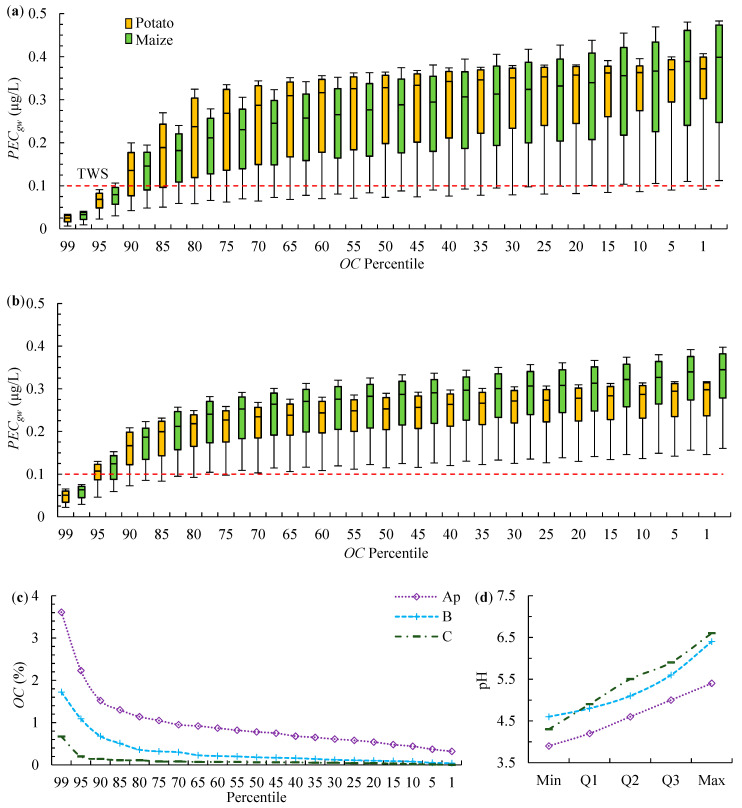
Predicted values of *PEC_gw_* for potato and maize cultivation in the model of AR profiles with varied *OC* content and pH for: (**a**) Lublin and (**b**) Hamburg climate. Box and whiskers plots denote the values of *PEC_gw_* estimated for the respective percentiles of *OC* content at minimum, first quartile (Q1), median (Q2), third quartile (Q3), and maximum pH values. (**c**) Percentiles of *OC* contents and (**d**) range of pH values of AR profiles.

A total of 255 AR profiles were selected from the database of Polish arable mineral soils [23] for this purpose. The percentiles (1st to 99th) of *OC* contents were calculated for the selected dataset (Table S5 in the Supplementary Materials). Next, *K_d_* values for each *OC* were calculated using Equation (1). The minimum, first quartile (Q1), median, third quartile (Q3), and maximum pH values (Figure 3), as well as the mean *Sand* content of 87.8%, 94.4%, and 96.4% for topsoils and two subsoils were calculated for Equation (1) based on the data from Table S1 in the Supplementary Materials. The calculated values of *K_d_*, 1/n *=* 0.95, as well as *OC*, *Sand*, and *Clay* contents (the mean values for topsoil and subsoils were 2.2%, 1.5%, and 1.7%) were used to simulations with model AR profiles with varied *OC* contents and pH. The applied degradation parameters were: *DT50* = 6.2 d, and *DDF* of 1:0.42:0.11.

The obtained values of *PEC_gw_* exceeded TGS in 77% of maize simulations, and in 70% of potato simulations. Values of *PECgw* were below TGS only in soils with the highest *OC* content (≥95th percentile) and in soils with the lowest pH (Figure 3a). The most probable range of *PEC_gw_* obtained for the Q1–Q3 range of the *OC* and pH was 0.136–0.375 μg/L for both crops.

The same simulations were performed using climate files from the Hamburg scenario (the Hamon equation was used). Such simulations (Figure 3b) can be representative of north-western and south-eastern Poland (Figure S5 in the Supplementary Materials), where precipitation is similar to the Hamburg scenario, as well as all of Poland as regards hot and rainy years. The AR soil group is highly prevalent in north-western Poland and less prevalent in south-eastern Poland [36]. The group, which covers 3.6% of the total land area in the EU, is also prevalent in Sweden, Denmark, and northern Germany. It is encountered on a smaller scale in England, northern France, Belgium, the Netherlands, Lithuania, and Latvia [36]. According to FOCUS, the Hamburg climate is largely representative of all of the above locations, excluding northern and central Sweden [4]. 

In most cases, the *PEC_gw_* range was narrower in Figure 3b than in Figure 3a (compare the ranges of error bars), and the values for the 99th–90th percentiles of *OC* content were higher in Figure 3b. For most profiles, TGS were exceeded even in soils characterized by the highest sorption at the lowest pH. The values of *PEC_gw_* obtained for the Q1–Q3 range of *OC* and pH values (0.175–0.287 μg/L for both crops) were within the range noted in the Lublin climate. Therefore, the leaching potential of AR soil group was similar in both examined climates. The simulations presented in Figure 3b indicate that when bentazone is applied in AR profiles with a very low pH, *PECgw* can be expected to exceed TGS in profiles where *OC* ≤ 1.3%, 0.5%, and 0.1% in topsoils and two subsoils, and in the case of a high soil pH in profiles with *OC* ≤ 3.0%, 1.4%, and 0.4%, respectively.

**Figure 4:**
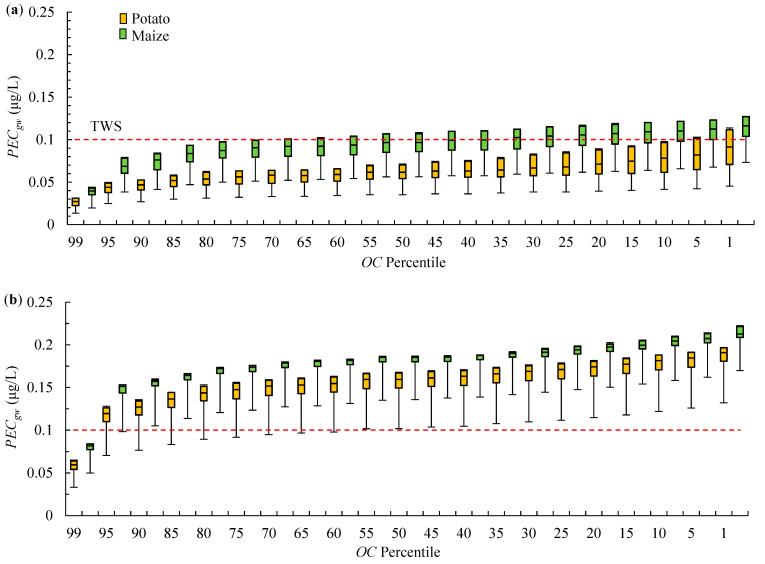

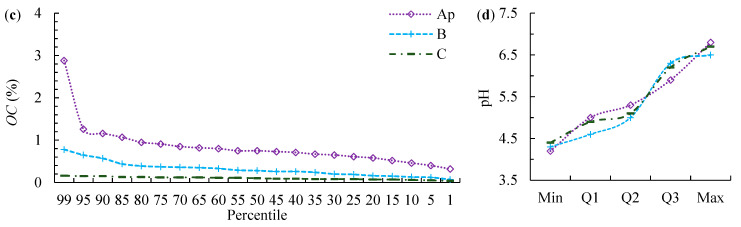
Predicted values of *PEC_gw_* for potato and maize cultivation in the model of LV profiles with varied *OC* content and pH for: (**a**) Lublin and (**b**) Hamburg climate. Box and whiskers plots denote the values of *PEC_gw_* estimated for the respective percentiles of *OC* content at minimum, first quartile (Q1), median (Q2), third quartile (Q3), and maximum pH values. (**c**) Percentiles of *OC* contents and (**d**) range of pH values of LV profiles.

Similar model profiles were created for the LV soil group. In this case, the percentiles of *OC* content were calculated based on data from 150 profiles of LV developed from loamy sand or sandy loam [23]. The mean values of *Sand* were 71.5%, 61.9%, and 52.3% for topsoils and two subsoils (Table S1 in the Supplementary Materials), and the mean values of *Clay* were 6.3%, 15.7%, and 23.5%, respectively. The simulations of the Lublin climate revealed that *PEC_gw_* exceeded TGS in 4% of the cases for potatoes and in 37% of the cases for maize (Figure 4a). An analysis of the water and pesticide mass balance suggested that higher values of *PEC_gw_* for maize resulted mainly from default settings affecting *ET* (458.1 L/m^2^/year for maize and 440.7 L/m^2^/year for potatoes), and bentazone uptake by plants (23.4–29.7% of the applied dose for maize, and 25.9–32.0% for potatoes). In maize, *PEC_gw_* exceeded TGS only in profiles with *OC* contents ≤ 75th percentile as well as medium or high soil pH. The most probable range of *PEC_gw_* for LV, obtained based on the Q1–Q3 range of *OC* and pH, was 0.048–0.116 μg/L.

As LV soil group is most prevalent in both north-western and south-eastern Poland, simulations involving the Hamburg climate were also carried out. It should also be noted that the LV group covers 15% of the land area in the EU and is found in nearly all European countries. Except Poland, in central and central-western Europe LV developed from loamy sand or sandy loam occur in northern Germany, Denmark, northern France, England, Lithuania, and Latvia [4,36]. As already mentioned, the Hamburg climate is largely representative of the above locations. 

The estimated *PEC_gw_* were much higher for both crops. The values of *PEC_gw_* were less likely to exceed TGS only in soils with the 99th percentile of *OC* content. In soils with *OC* contents of the 95th percentile or lower, *PEC_gw_* exceeded TGS within nearly the entire examined pH range, excluding several profiles with the highest *OC* and the lowest pH values for potatoes. The values of *PEC_gw_* for the Q1–Q3 range of *OC* and pH values were determined at 0.137–0.198 μg/L for both crops. Data presented in Figure 4b indicate that when bentazone is used in acidic soils, values of *PECgw* are likely to exceed TGS in profiles with *OC* ≤ 1.3%, 0.7%, and 0.2% in topsoils and two subsoils, and when bentazone is applied in soils with a high pH, values of *PECgw* are likely to exceed TGS in profiles with *OC* ≤ 2.5%, 0.8%, and 0.2%, respectively.

The above findings are consistent with the results obtained by Lammoglia et al. [21], who observed that the values of *PEC_gw_* of bentazone in profile with the texture of clay loam to silty clay loam were affected mainly by differences in annual precipitation. Rasmussen et al. [37] examined the impact of rainfall intensity on pesticide leaching and found that rainfall significantly contributed to bentazone leaching in sandy loam soil, whereas no differences were noted in sandy soil. Therefore, the observed differences in bentazone leaching in AR and LV profiles in Lublin and Hamburg climates could be attributed to the higher water-holding capacity of LV, which to some extent prevented bentazone leaching in this soil group in the Lublin climate, but not in AR profiles. 

The simulations presented in Figure 3 and Figure 4 indicate that low soil pH, which is detrimental to plant cultivation, can greatly reduce bentazone leaching to groundwater. However, in AR profiles, and in LV profiles in simulations of the Hamburg climate, bentazone sorption was generally too low to prevent exceeding TGS even in acidic soils. Moreover, a comparison of the most probable ranges of *PEC_gw_* based on the Q1–Q3 range of *OC* and pH values with the values obtained for the Hamburg scenario (Figure 1) confirmed that the Hamburg scenario is not representative of AR and LV soil groups in both examined climates.

The values of *PEC_gw_* presented in Figure 3b and Figure 4b are lower than those obtained in the modeling study by Tiktak et al. [32]. The cited authors estimated that bentazone concentrations in groundwater were highest in Dutch soils with the texture of sand or loamy sand, reaching 1 μg/L in soils with low *OC* contents. However, they used more conservative settings for adsorption and degradation parameters (e.g., *DT50* = 16 d). In Denmark, Rosenbom et al. [18] monitored bentazone leaching to groundwater in the Jyndevad field (texture of sand, *OC* content in the arable layer—1.8%, pH 5.6–6.2) and in the Tylstrup field (texture of loamy sand, *OC*—2.0%, pH 4.0–4.5). Properties of the Jyndevad and Tylstrup soils are similar to the Polish AR and LV profiles. In Jyndevad, bentazone was detected in 32.5% of the samples with the use of suction cups placed 1 m b.g.s. In 4% of the samples, bentazone concentrations exceeded TGS, and the maximum concentration was 1.6 μg/L. In Tylstrup, bentazone was detected in one sample at a concentration of 0.01 μg/L. In view of the simulation results presented in Figure 3b, the predicted value of *PEC_gw_* for Jyndevad, estimated for both crops for the Hamburg climate as the average value the for 95th and 90th percentiles of *OC* at maximum pH, was 0.18 μg/L. The *PEC_gw_* predicted for Tylstrup, estimated for the Hamburg climate as the average value for the 99th and 95th percentiles of *OC* at minimum pH (Figure 4b), was 0.06 μg/L for both crops. Considering that climate data for the above locations were not used and that soil data were available only for the arable layer, the resulting predictions are quite accurate for both Jyndevad and Tylstrup.

## 4. Conclusions

The values of *PEC_gw_* for bentazone were highly sensitive to degradation parameters, organic matter content, and soil pH. Soil properties associated with water-holding capacity, water conductivity, and evapotranspiration also exerted a considerable impact on *PEC_gw_*. 

In the examined soil groups, values of *PEC_gw_* were highest in AR profiles, and only minimal differences were observed between results for the Lublin climate, which is characteristic of the Polish climate and the FOCUS Hamburg climate. Simulation results suggest that in the AR soil group the use of bentazone for crop protection should be limited to soils with high *OC* content (3.0% or higher for topsoil). The values of *PEC_gw_* in the LV soil group were largely dependent on climate conditions. In simulations based on the Lublin, climate *PEC_gw_* values were below TGS only in profiles with the lowest *OC* contents and the highest pH. This observation suggests that in the LV soil group, bentazone can be safely applied in regions with an estimated annual precipitation ≤600 mm. The simulations based on the Hamburg climate revealed that in areas with a mean annual precipitation ≥ 750 mm, bentazone can be safely applied only in soils with high *OC* content (2.5% or higher for topsoil). The simulations carried out in the LV&CM soil group revealed that TGS were unlikely to be exceeded in these soils with high water-holding capacity and high *OC* content.

A comparison of the obtained results with values noted in the FOCUS Hamburg scenario, which represents the coarsest soils in the EU with a relatively low *OC* contents, demonstrated that this scenario does not reflect the leaching potential of the examined AR and LV soil groups.

## Data Availability

All relevant data are within the text and the Supplementary Materials.

## Supplementary Materials

The following supporting information can be downloaded at: https://www.mdpi.com/article/10.3390/ijerph19127187/s1, Figure S1: Locations of the studied soil profiles on the map of Poland; Figure S2: Box and whiskers plots of *Sand*, *Clay*, *OC*, and pH in AR, LV, and LV&CM soil groups; Figure S3: Adsorption isotherms of bentazone in the six selected profiles of mineral soils; Figure S4: Comparison of the mean annual precipitation and temperature of the FOCUS Hamburg and Kremsmünster climates with the Lublin climate; Figure S5: Mean annual temperature and precipitation in Poland; Figure S6: PCA results: (a) The effect of soil properties on *PEC_gw_* in AR, LV, and LV&CM soil groups in simulations of maize cultivation; (b) The effect of soil properties on *PEC_gw_* in the AR soil group in simulations of maize cultivation; Figure S7: Comparison of means, standard deviations, and results of ANOVA analyses for the selected soil properties; Figure S8: Relationships between *PEC_gw_* and *Ks*, and between *PEC_gw_* and *FC* for potato and maize cultivation; Table S1: Basic physical and chemical properties and locations of soils from 30 profiles of AR, LV, and LV&CM soil groups; Table S2: Results of fitting of the Freundlich equation to data from the batch adsorption experiments: Table S3: Soil properties for Hamburg scenario; Table S4: Values of *p* for Levene’s tests, ANOVA F and post hoc LSD tests, or Kruskal–Wallis and post hoc Dunn’s tests for the selected soil properties; Table S5: Percentiles of *OC* contents in the AR and LV soil groups according to the database and soil collection of the Institute of Agrophysics of the Polish Academy of Sciences; References [38,39] are cited in the Supplementary Materials.

## References

[1] European Commission (2006). Directive 2006/118/EC of the European Parliament and of the Council of 12 December 2006 on the protection of groundwater against pollution and deterioration. Off. J..

[2] Jørgensen L.F., Stockmarr J. (2009). Groundwater monitoring in Denmark: Characteristics, perspectives and comparison with other countries. Hydrogeol. J..

[3] European Commission (2009). Regulation (EC) No 1107/2009 of the European Parliament and of the Council of 21 October 2009 concerning the placing of plant protection products on the market and repealing Council Directives 79/117/EEC and 91/414/EEC. Off. J..

[4] European Commission (2014). Assessing Potential for Movement of Active Substances and Their Metabolites to Ground Water in the EU. Report of the FOCUS Ground Water Work Group.

[5] EU Pesticides Database. http://ec.europa.eu/food/plant/pesticides/eu-pesticides-database/public/?event=homepage&language=EN.

[6] Comer J., Chamberlain K., Evans A. (1995). Validation of pH-metric technique for measurement of pKa and log Pow of ionizable herbicides. SAR QSAR Environ. Res..

[7] Paszko T., Matysiak J., Kaminski D., Pasieczna-Patkowska S., Huber M., Krol B. (2020). Adsorption of bentazone in the profiles of mineral soils with low organic matter content. PLoS ONE.

[8] EFSA (2015). Conclusion on the peer review of the pesticide risk assessment of the active substance bentazone. EFSA J..

[9] Hvĕzdová M., Kosubová P., Košiková M., Scherr K.E., Šimek Z., Brodský L., Šudoma M., Škulcová L., Sáňka M., Svobodová M. (2018). Currently and recently used pesticides in Central European arable soils. Sci. Total Environ..

[10] Tauchnitz N., Kurzius F., Rupp H., Schmidt G., Hauser B., Schrödter M., Meissner R. (2020). Assessment of pesticide inputs into surface waters by agricultural and urban sources—A case study in the Querne/Weida catchment, central Germany. Environ. Pollut..

[11] Weber G., Christmann N., Thiery A.C., Martens D., Kubiniok J. (2018). Pesticides in agricultural headwater streams in southwestern Germany and effects on macroinvertebrate populations. Sci. Total Environ..

[12] Diamanti K.S., Alygizakis N.A., Nika M.C., Oswaldova M., Oswald P., Thomaidis N.S., Slobodnik J. (2020). Assessment of the chemical pollution status of the Dniester River Basin by wide-scope target and suspect screening using mass spectrometric techniques. Anal. Bioanal. Chem..

[13] Loos R., Gawlik B.M., Locoro G., Rimaviciute E., Contini S., Bidoglio G. (2009). EU-wide survey of polar organic persistent pollutants in European river waters. Environ. Pollut..

[14] McManus S.L., Richards K.G., Grant J., Mannix A., Coxon C.E. (2014). Pesticide occurrence in groundwater and the physical characteristics in association with these detections in Ireland. Environ. Monit. Assess..

[15] Loos R., Locoro G., Comero S., Contini S., Schwesig D., Werres F., Balsaa P., Gans O., Weiss S., Blaha L. (2010). Pan-European survey on the occurrence of selected polar organic persistent pollutants in ground water. Water Res..

[16] Åkesson M., Sparrenbom C.J., Dahlqvist P., Fraser S.J. (2015). On the scope and management of pesticide pollution of Swedish groundwater resources: The Scanian example. Ambio.

[17] Kock-Schulmeyer M., Ginebreda A., Postigo C., Garrido T., Fraile J., López de Alda M., Barceló D. (2014). Four-year advanced monitoring program of polar pesticides in groundwater of Catalonia (NE-Spain). Sci. Total Environ..

[18] Rosenbom A.E., Olsen P., Plauborg F., Grant R., Juhler R.K., Brüsch W., Kjær J. (2015). Pesticide leaching through sandy and loamy fields—Long-term lessons learnt from the Danish Pesticide Leaching Assessment Programme. Environ. Pollut..

[19] Schuhmann A., Klammler G., Weiss S., Gans O., Frank J., Haberhauer G., Gerzabek M.H. (2019). Degradation and leaching of bentazone, terbuthylazine and S-metolachlor and some of their metabolites: A long-term lysimeter experiment. Plant Soil Environ..

[20] Lewis K.A., Tzilivakis J., Warner D.J., Green A. (2016). An international database for pesticide risk assessments and management. Hum. Ecol. Risk Assess..

[21] Lammoglia S.K., Brun F., Quemar T., Moeys J., Barriuso E., Gabrielle B., Mamy L. (2018). Modelling pesticides leaching in cropping systems: Effect of uncertainties in climate, agricultural practices, soil and pesticide properties. Environ. Modell. Softw..

[22] Tiktak A. (2000). Application of pesticide leaching models to the Vredepeel dataset: II Pesticide fate. Agric. Water Manag..

[23] Bieganowski A., Witkowska-Walczak B., Gliński J., Sokołowska Z., Sławiński C., Brzezińska M., Włodarczyk T. (2013). Database of Polish arable mineral soils: A review. Int. Agrophys..

[24] WRB (2015). World Reference Base for Soil Resources 2014, Update 2015. International Soil Classification System for Naming Soils and Creating Legends for Soil Maps. World Soil Resources Reports No. 106.

[25] Paszko T., Muszyński P. (2017). Degradation rates of alachlor, atrazine and bentazone in the profiles of Polish Luvisols. Int. Agrophys..

[26] OECD (2000). OECD Guideline for the Testing of Chemicals. Method 106. Adsorption-Desorption Using Batch Equilibrium Method.

[27] Klein M. (2018). PELMO (Pesticide Leaching Model). Version 5.00. User Manual.

[28] Statistics Poland (2020). Statistical Yearbook of Agriculture.

[29] Paszko T. (2014). Adsorption, degradation and mobility of carbendazim in profiles of Polish mineral soils. Geoderma.

[30] Paszko T. (2014). Modeling of pH-dependent adsorption and leaching of MCPA in profiles of Polish mineral soils. Sci. Total Environ..

[31] Rodríguez Cruz M.S., Jones J.E., Bending G.D. (2008). Study of the spatial variation of the biodegradation rate of the herbicide bentazone with soil depth using contrasting incubation methods. Chemosphere.

[32] Tiktak A., van der Linden A.M.A., Leine I., Corwin D.L., Loague K. (1996). Application of GIS to the modeling of pesticide leaching on a regional scale in the Netherlands. Application of GIS to the Modeling of Non-Point Source Pollutants in the Vadose Zone.

[33] Boesten J.J.T.I., van der Pas L.J.T. (2000). Movement of water, bromide and the pesticides ethoprophos and bentazone in a sandy soil: The Vredepeel data set. Agric. Water Manag..

[34] Spencer W.F., Cliath M.M. (1973). Pesticide volatilization as related to water loss from soil. J. Environ. Qual..

[35] Wolters A., Klein M., Vereecken H. (2004). An improved description of pesticide volatilization: Refinement of the pesticide leaching model (PELMO). J. Environ. Qual..

[36] Tόth G., Montanarella L., Stolbovoy V., Máté F., Bόdis K., Jones A., Panagos P., Van Liedekerke M. (2008). Soils of the European Union.

[37] Rasmussen S.B., Abrahamsen P., Nielsen M.H., Holm P.E., Hansen S. (2015). Effects of single rainfall events on leaching of glyphosate and bentazone on two different soil types, using the DAISY model. Vadose Zone J..

[38] FOCUS FOCUS Groundwater Scenarios in the EU Review of Active Substances.

[39] (2009). Soil Quality—Determination of Particle Size Distribution in Mineral Soil Material—Method by Sieving and Sedimentation.

