# Supplementary material for: Modeling of Bentazone Leaching in Soils with Low Organic Matter Content
Source: Int J Environ Res Public Health. 2022 Jun 11;19(12):7187. doi: 10.3390/ijerph19127187 (PMC9223228; doi:10.3390/ijerph19127187)
Supplement: Supplementary file 1 [file ijerph-19-07187-s001.zip › ijerph-1647697-supplementary.pdf]

Supplementary Materials for:

Modelling of bentazone leaching in soils with low organic matter content

Tadeusz Paszko[1]*, Claudio Aparecido Spadotto[2]

[1] *Department of Chemistry, University of Life Sciences, Akademicka 13, 20-950 Lublin, Poland*

[2]*Embrapa Digital Agriculture, Av. André Tosello, 209,Campinas (SP) 13083-886, Brazil*

**Corresponding author*. Tel. +48 81 445 6640

E-mail address: tadeusz.paszko@up.lublin.pl

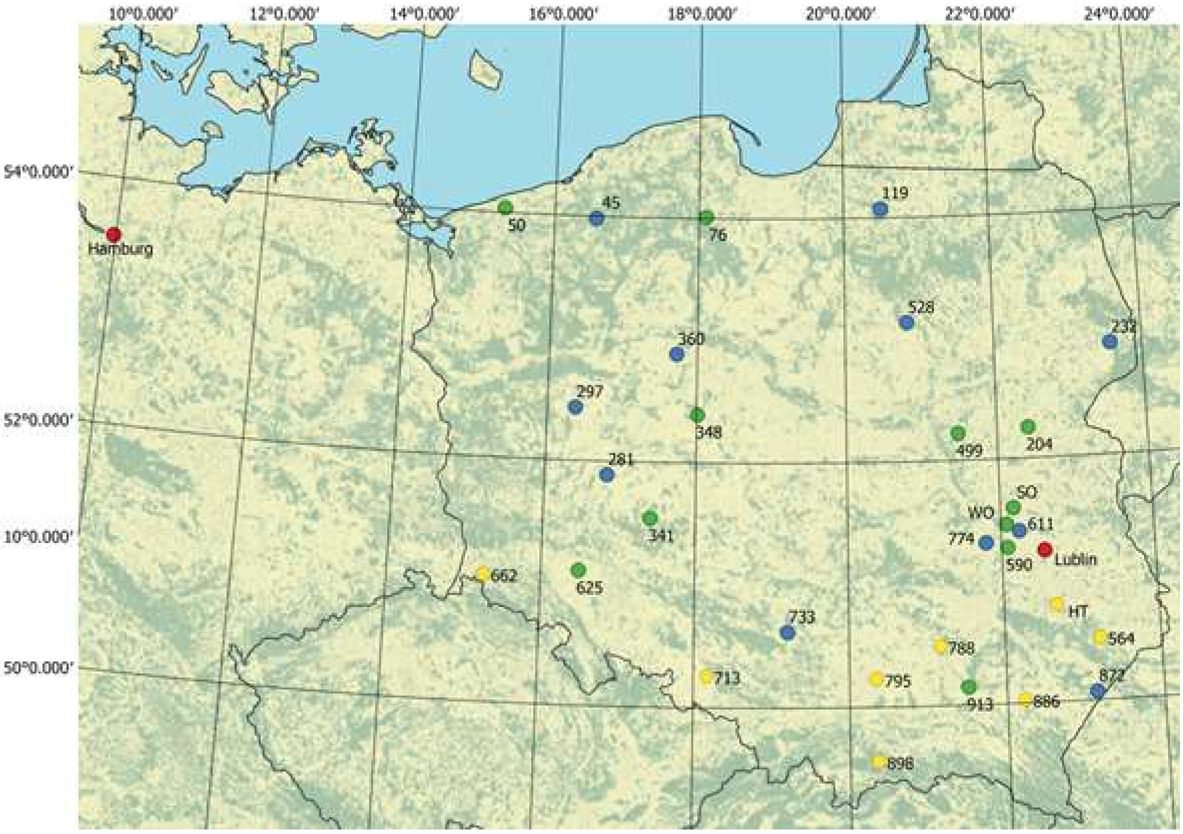

Figure. S1. Locations of the studied soil profiles (Arenosols, Luvisols, Luvisols and Cambisols) on the map of Poland and sources of the examined climatic data ( ).

Table S1. Basic physical and chemical properties and locations of soils from 30 profiles of AR, LV and LV&CM soil groups.

Code	Location	Hor.	Depth (cm)	*Sand*[a] (%) 2000-50	*Silt*[a] (%) 50-2	*Clay*[a] (%) < 2	*OC*[b] (%)	pH ($CaCl_2$)	*FC*[c] (L/L)	*Ks*[d] (cm/day)
				**Arenosols (AR)**						
45	Gołdaw[e]	Ap	10-15	82.2	15.4	2.4	1.13	5.0	0.240	111.6
	(53°59′N, 16°34′E)	BC	35-40	93.9	4.8	1.3	0.10	5.8	0.215	419.2
		C	90-95	93.5	4.8	1.7	0.02	6.0	0.216	299.7
119	Wróblik[e]	Ap	20-25	90.2	7.0	2.8	1.42	4.6	0.225	291.8
	(54°04′N, 20°27′E)	Bw	60-65	96.5	1.9	1.6	0.33	5.5	0.210	600.1
		C	100-105	97.5	1.0	1.5	0.06	5.5	0.208	555.0
232	Makówka[e]	Ap	10-20	92.7	5.4	1.9	0.88	4.2	0.218	435.7
	(52°54′N, 23°33′E)	Bw	35-45	98.1	1.1	0.8	0.35	5.1	0.205	785.4
		C	90-100	98.7	0.4	0.9	0.00	5.5	0.204	686.4
281	Górzno[e]	Ap	10-20	87.8	9.1	3.1	1.06	3.9	0.231	207.1
	(51°53′N, 16°49′E)	Bw	40-50	94.5	4.7	0.8	0.31	4.8	0.213	470.7

		C	90-100	95.4	2.6	2.0	0.07	5.2	0.213	392.8
297	Śliwno[e]	Ap	0-30	83.9	14.3	1.8	1.09	4.7	0.236	137.7
	(52°26´N, 16°22´E)	BC	30-50	88.2	9.6	2.2	0.10	5.6	0.228	174.1
		C	80-100	94.9	3.2	1.9	0.05	5.8	0.214	366.5
360	Dobrylewo[e]	Ap	10-20	92.1	5.1	2.8	0.69	5.2	0.221	381.7
	(52°53´N, 17°43´E)	BC	40-50	96.9	1.6	1.5	0.08	6.4	0.209	638.6
		C	100-110	97.9	0.3	1.8	0.00	6.6	0.208	577.0
528	Marianowo[e]	Ap	10-25	84.4	13.8	1.8	1.25	5.4	0.235	145.7
	(53°08´N, 20°49´E)	BC	40-60	94.1	3.8	2.1	0.09	4.8	0.216	412.3
		C	70-90	97.5	0.7	1.8	0.05	5.4	0.209	544.3
611	Olempin[e]	Ap	5-10	87.5	10.1	2.4	0.68	4.1	0.230	206.4
	(51°24´N, 22°14´E)	BC	35-45	92.6	5.7	1.7	0.09	4.6	0.218	338.2
		C	65-75	96.1	2.4	1.5	0.03	4.3	0.211	450.8
733	Rosochacz[e]	Ap	0-25	83.3	14.8	1.9	1.30	4.9	0.237	128.4
	(50°37´N, 19°12´E)	Bw	25-60	92.0	5.8	2.2	0.48	4.6	0.220	300.8
		C	60-150	94.3	3.8	1.9	0.06	4.5	0.215	334.5
774	Jedlińsk[e]	Ap	10-20	89.3	8.7	2.0	1.11	4.4	0.225	268.3
	(51°32´N, 21°08´E)	Bw	40-50	95.1	3.9	1.0	0.30	5.1	0.212	508.0
		C	105-110	96.9	1.0	2.1	0.00	6.1	0.210	488.5
872	Łukawiec[e]	Ap	10-20	92.1	6.1	1.8	0.80	4.2	0.219	402.2
	(50°04´N, 23°08´E)	Bw	60-70	96.3	2.0	1.7	0.17	4.7	0.211	580.0
		C	100-110	98.0	0.8	1.2	0.03	4.4	0.206	608.6
**Luvisols (LV)**										
50	Niemierze[e]	Ap	5-10	71.5	21.8	6.7	1.23	5.2	0.270	37.1
	(54°04´N, 15°31´E)	E	30-35	71.2	21.5	7.3	0.35	4.5	0.272	35.3
		Bt	90-95	61.0	21.5	17.5	0.15	5.3	0.313	9.6
76	Wilcze Błota[e]	Ap	10-30	73.3	18.6	8.1	1.23	5.2	0.270	38.0
	(54°01´N, 18°10´E)	Bt1	40-60	55.4	22.9	21.7	0.18	4.6	0.333	8.8
		Bt2	80-100	59.3	18.6	22.1	0.11	4.5	0.326	7.2
204	Drohiczyn[e]	Ap	5-15	71.6	20.9	7.5	1.69	6.1	0.271	35.7
	(52°25´N, 22°41´E)	E	30-40	76.4	17.9	5.7	0.36	6.3	0.259	53.0
		Bt	100-110	49.7	25.5	24.8	0.08	6.7	0.350	4.4
341	Fabianów[e]	Ap	10-20	68.9	21.7	9.4	1.16	6.8	0.281	28.1
	(51°53´N, 17°41´E)	Bt1	40-45	67.7	18.1	14.2	0.26	6.5	0.293	20.6
		Bt2	100-110	56.4	21.9	21.7	0.13	6.0	0.331	6.6
348	Olsza[e]	Ap	10-30	86.3	9.8	3.9	0.72	5.7	0.235	152.8
	(52°39´N, 18°01´E)	E	30-50	78.2	16.5	5.3	0.13	6.4	0.254	62.1
		Bt	80-100	60.2	21.3	18.5	0.09	6.3	0.317	8.9
499	Pniewnik[e]	Ap	0-25	80.7	14.4	4.9	1.29	4.2	0.248	78.8
	(52°22´N, 21°48´E)	E	25-45	72.4	18.8	8.8	0.20	4.5	0.273	34.7

		Bt	45-110	59.1	23.8	17.1	0.16	4.9	0.316	9.2
590	Dęba[e]	Ap	5-15	80.0	16.5	3.5	0.93	4.7	0.247	79.6
	(51°26´N, 22°10´E)	E	35-45	79.8	15.3	4.9	0.12	5.0	0.250	72.6
		Bt	65-75	60.0	20.6	19.4	0.08	4.9	0.319	8.4
625	Krępice[e]	Ap	10-20	69.1	22.6	8.3	1.17	5.3	0.278	30.1
	(51°10´N, 16°49´E)	Bt	50-55	42.0	30.0	28.0	0.15	6.3	0.372	3.9
		BC	110-115	49.8	27.6	22.6	0.07	5.0	0.346	4.9
913	Werynia[e]	Ap	5-20	72.0	20.9	7.1	0.91	4.4	0.270	37.2
	(50°15´N, 21°52´E)	Bt	40-50	50.6	23.4	26.0	0.15	4.3	0.351	6.2
		BC	80-90	56.6	23.9	19.5	0.06	5.1	0.326	7.4
WO	Wola Osińska[f]	Ap	5-20	60.0	35.0	5.0	0.75	5.4	0.290	26.9
	(51°46´N, 22°10´E)	Bt1	30-50	41.0	31.0	28.0	0.26	4.7	0.374	3.8
		Bt2	70-80	24.0	30.0	46.0	0.12	4.4	0.444	2.3
SO	Sobieszyn[f]	Ap	5-15	53.0	42.0	5.0	0.80	6.7	0.304	22.6
	(51°60´N, 22°17´E)	Bt1	30-40	46.0	31.0	23.0	0.28	6.2	0.354	5.5
		Bt2	55-60	39.0	32.0	29.0	0.10	6.4	0.380	2.5
**Luvisols or Cambisols (LV&CM)**										
564	Skierbieszów[e]	Ap	10-20	25.5	64.8	9.7	1.23	6.6	0.368	30.5
	(50°51´N, 23°22´E)	Bw	35-50	18.5	71.7	9.8	0.53	6.3	0.383	23.0
		BC	65-75	21.3	68.3	10.4	0.49	6.2	0.378	18.1
662	Ubocze[e]	Ap	15-25	16.0	80.4	3.6	1.80	7.2	0.375	51.4
	(51°04´N, 15°26´E)	Bw	35-45	21.3	74.5	4.2	0.99	5.5	0.366	36.7
		Bt	100-110	26.0	63.9	10.1	0.31	5.3	0.368	17.9
713	Klucz[e]	Ap	0-35	16.6	81.3	2.1	1.33	6.8	0.371	57.4
	(50°26´N, 18°17´E)	Bt1	35-75	18.4	67.9	13.7	0.24	6.2	0.391	16.4
		Bt2	75-150	14.9	69.2	15.9	0.13	5.1	0.402	11.8
788	Łężyce[e]	Ap	10-20	16.8	73.2	10.0	1.05	7.1	0.386	31.8
	(50°49´N, 21°20´E)	Bw	25-35	21.5	67.3	11.2	0.56	7.3	0.379	19.8
		BC_{ca}	110-120	16.3	77.4	6.3	0.46	7.6	0.380	26.2
795	Złota[e]	Ap	10-20	14.6	78.5	6.9	1.02	5.1	0.385	40.1
	(50°23´N, 20°34´E)	Bw	30-40	22.0	69.1	8.9	0.27	6.4	0.374	24.3
		BC_{ca}	110-120	19.6	73.8	6.6	0.47	7.7	0.374	25.5
886	Studzian[e]	Ap	5-15	15.1	74.6	10.3	1.27	5.2	0.390	31.1
	(50°03´N, 22°22´E)	Bt	50-60	22.7	61.5	15.8	0.20	6.2	0.386	13.0
		BC	110-120	22.6	68.1	9.3	0.12	6.6	0.373	19.8
898	Uszwica Porębska[e]	Ap	15-25	13.0	80.7	6.3	1.39	5.1	0.387	41.3
	(49°56´N, 20°35´E)	Bt	50-60	17.5	62.8	19.7	0.24	4.9	0.404	10.1
		BC	90-100	14.5	71.5	14.0	0.13	5.1	0.399	13.9
HT	Huta Turobińska[f]	Ap	5-15	15.0	80.0	5.0	0.76	6.1	0.380	46.2
	(50°80´N, 22°69´E)	E	25-35	22.0	76.0	2.0	0.5	5.4	0.360	44.5

	Bt	75-90	12.0	71.0	17.0	0.07	4.7		0.410	10.9

[a] soil texture - determined using the pipette method (ISO 11277, 2009); [b] organic carbon content - determined using a Shimadzu TOC-VCSH analyser and a SSM-5000A solid sample module; [c] water content at field capacity - estimated using pedotransfer functions of PELMO (Klein, 2018); [d] saturated hydraulic conductivity - estimated using Rosetta Version 1.2; [e] profiles selected and soil samples obtained from the database and soil collection of the Institute of Agrophysics of the Polish Academy of Science in Lublin (Bieganowski et al., 2013); [f] profiles selected based on recommendations from the Institute of Soil Sciences and Environment Shaping of the University of Life Sciences in Lublin.

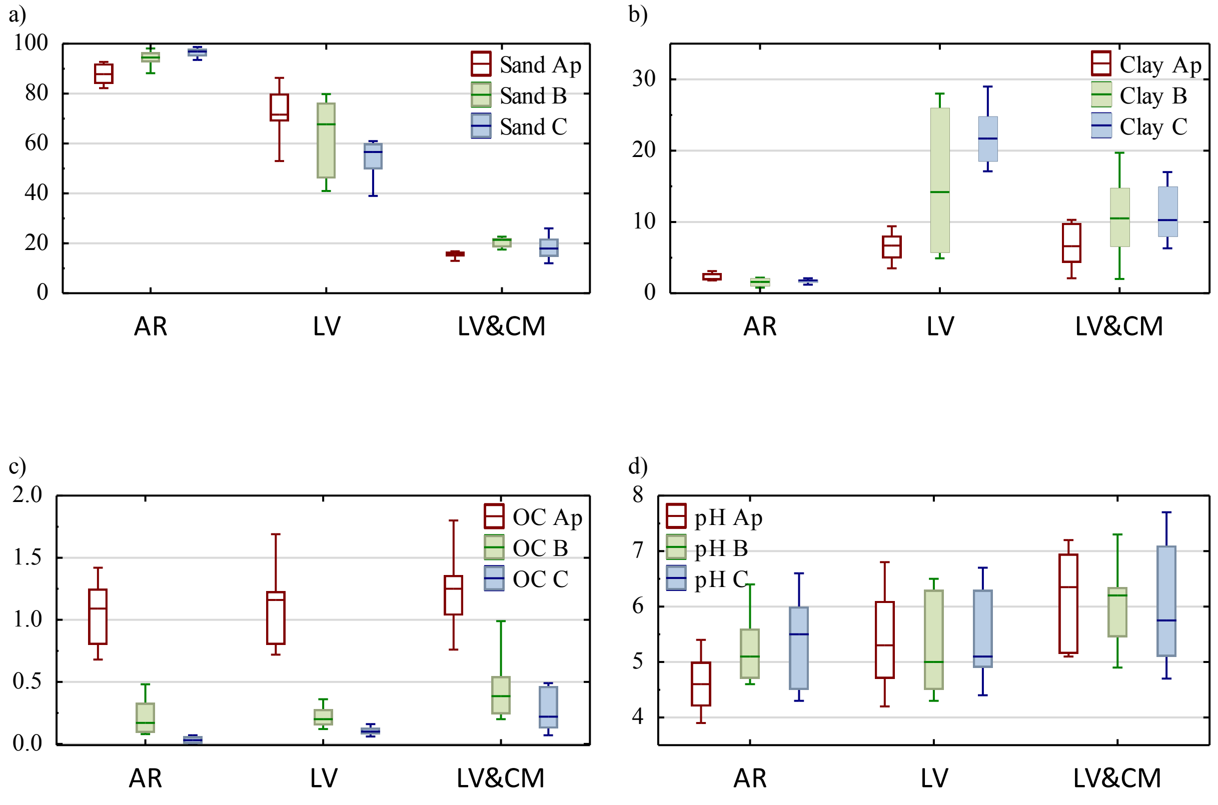

Figure. S2. Box and whiskers plots (minimum, Q1, median, Q3 and maximum) of *Sand*, *Clay*, *OC*, and pH in AR, LV and LV&CM soil groups.

The used form of the Freundlich model that was fitted to data from adsorption experiments was:

$$S_{ads} = K_F \cdot C_{aq}(\mathrm{eq})^{1/n} \quad \text{(S1)}$$

where S_{ads} (µg/g) and C_{aq}(eq) (µg/mL) are concentrations of the test substance at adsorption equilibrium in the solid and liquid phase, respectively, K_F (µg $^{1-1/n}$ (mL)$^{1/n}$ g^{-1}) is the Freundlich adsorption coefficient, and *1/n* is the Freundlich exponent. Results are visualized in Fig. S3 and the obtained Freundlich coefficients are listed in Table S2. Adsorption isotherms were determined in the HT, WO and SO profiles of LV used previously for degradation experiments (Paszko and Muszyński, 2017), and additionally in the 611, 590 and 564 profiles representing the typical AR, LV, and LV&CM profiles (Table S1).

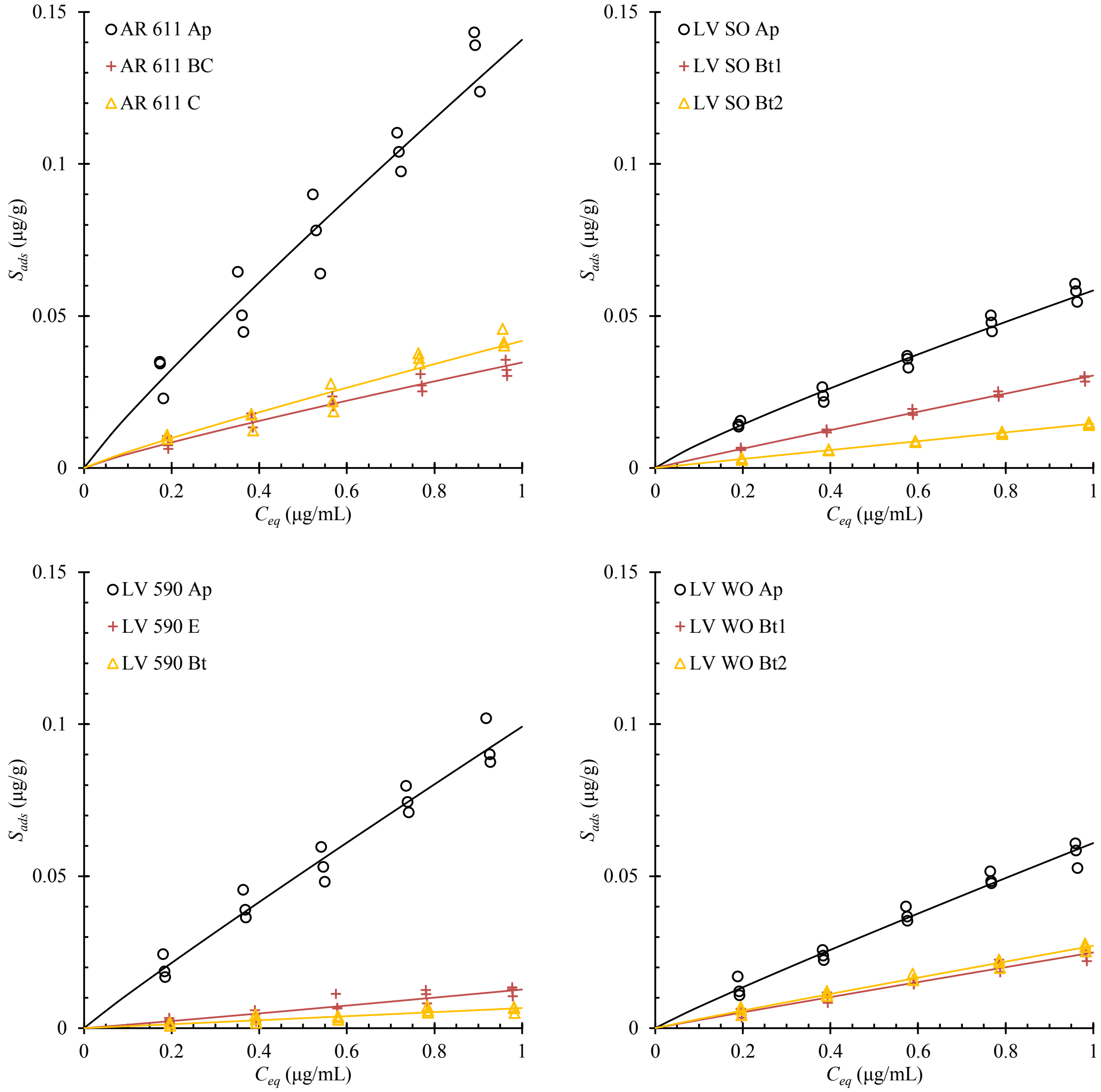

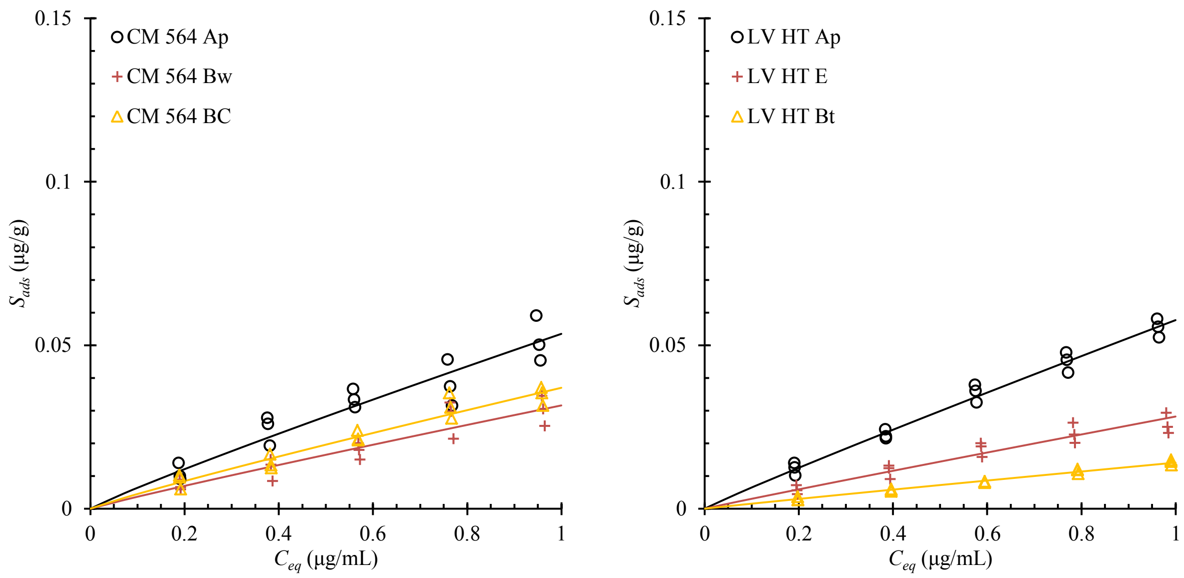

Figure. S3. Adsorption isotherms of bentazone in the six selected profiles of mineral soils. Lines represent results of fitting of the Freundlich equation (for details see Table S2).

Table S2. Results of fitting of the Freundlich equation to data from the batch adsorption experiments presented in Fig. S3.

Soil	K_F (µg$^{1-1/n}$ (mL)$^{1/n}$ g^{-1})	*1/n*	R^2	K_{FOC} (µg$^{1-1/n}$ (mL)$^{1/n}$ g^{-1})
AR 611 Ap	0.141	0.91	0.942	20.7
AR 611 BC	0.035	0.88	0.923	38.9
AR 611 C	0.042	0.90	0.940	140.0
LV SO Ap	0.058	0.87	0.982	7.3
LV SO Bt1	0.032	0.97	0.995	11.4
LV SO Bt2	0.015	0.99	0.993	15.0
LV 590 Ap	0.099	0.95	0.963	10.6
LV 590 E	0.013	1.05	0.821	10.8
LV 590 Bt	0.007	1.01	0.787	8.8
LV WO Ap	0.061	0.94	0.963	8.1
LV WO Bt1	0.025	0.97	0.921	9.6
LV WO Bt2	0.027	0.96	0.965	22.5
CM 564 Ap	0.054	0.92	0.870	4.4

CM 564 Bw	0.032	0.94	0.886	6.0
CM 564 BC	0.037	0.92	0.925	7.6
LV HT Ap	0.058	0.95	0.977	7.6
LV HT E	0.028	0.97	0.930	5.6
LV HT Bt	0.014	0.96	0.981	20.0

Table S3. Soil properties for Hamburg scenario (FOCUS, 2000).

Horizon	Depth (cm)	pH-H_2O	pH-KCl	Texture (mm) <0.002	0.002-0.05	>0.05	OC (%)	Bulk Density (g cm^{-3})	DDF
Ap	0-30	6.4	5.7	7.2	24.5	68.3	1.5	1.5	1
BvI	30-60	5.6	4.9	6.7	26.3	67	1	1.6	0.5
BvII	60-75	5.6	4.9	0.9	2.9	96.2	0.2	1.56	0.3
Bv/Cv	75-90	5.7	5.0	0	0.2	99.8	0	1.62	0.3
Cv	90-100	5.5	4.8	0	0	100	0	1.6	0.3
Cv	100-200	5.5	4.8	0	0	100	0	1.6	0

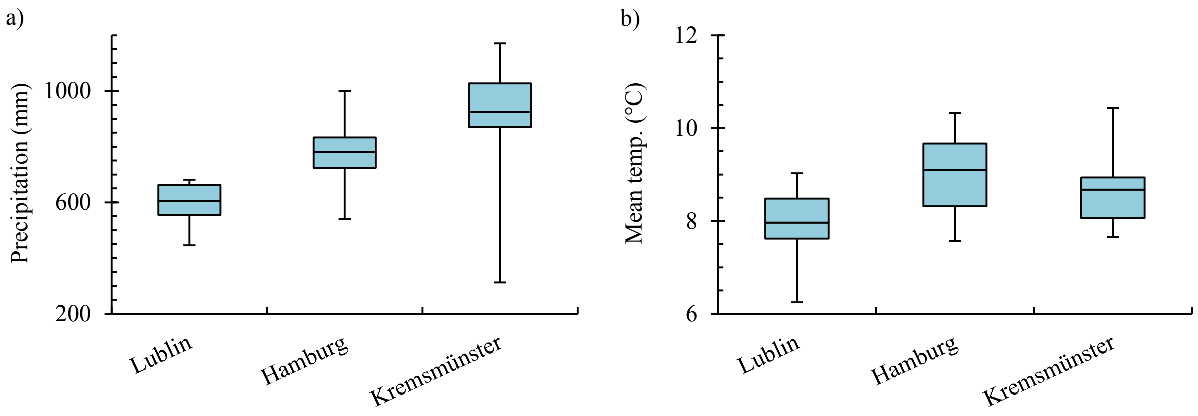

Figure. S4. Comparison of the mean annual precipitation and temperature of the FOCUS Hamburg and Kremsmünster climates with the Lublin climate, representing typical climate of Poland (FOCUS, 2000).

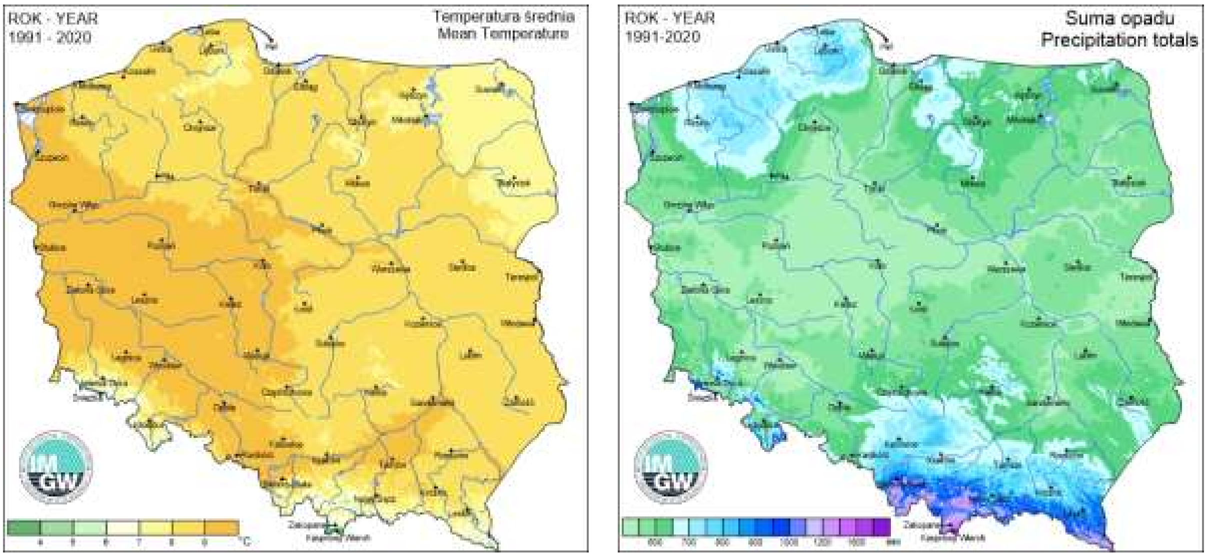

Figure. S5. Mean annual temperature and precipitation in Poland (1991-2020) according to the Institute of Meteorology and Water Management. Data from: https://klimat.imgw.pl/pl/climate-maps/#Precipitation/Yearly/1991-2020/1/Winter (accessed on 10 January 2022)

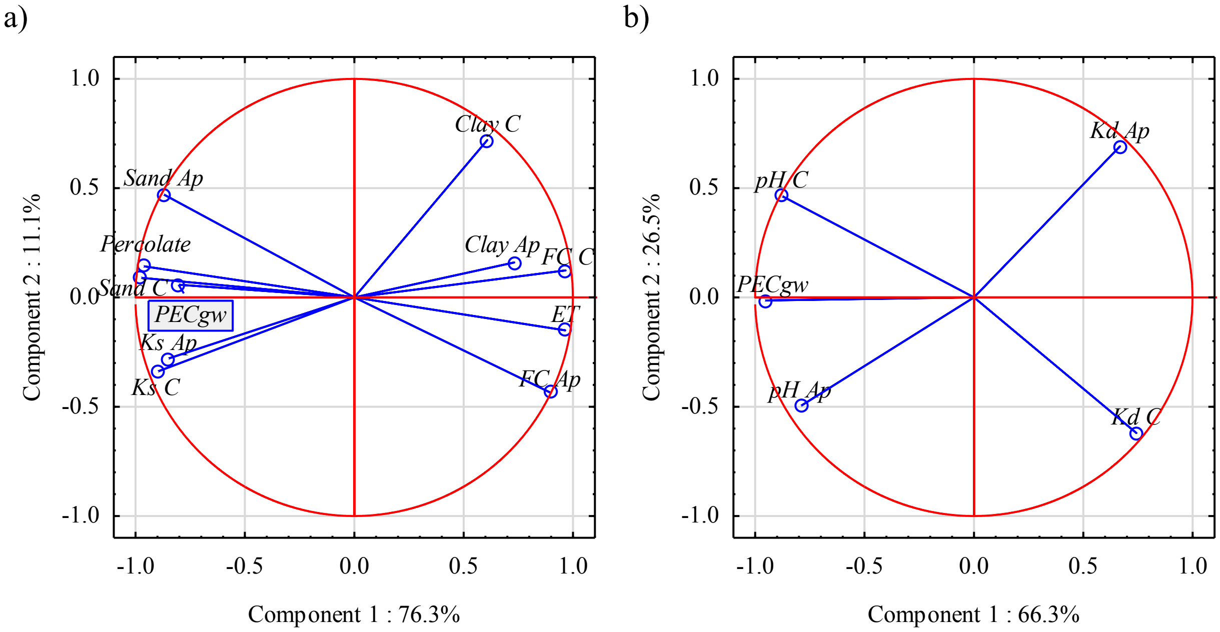

Figure. S6. PCA results: (a) The effect of soil properties on PEC_{gw} in AR, LV, and LV&CM soil groups in simulations of maize cultivation; (b) The effect of soil properties on PEC_{gw} in the AR soil group in simulations of maize cultivation.

Table S4. Values of *p* for Levene's tests, ANOVA F and post hoc LSD tests, or Kruskal-Wallis H and post hoc Dunn's tests for the selected soil properties.

Variable	Levene's test	ANOVA F test	Kruskal-Wallis H test	Post hoc LSD or Dunn's tests			
					AR	LV	LV&CM
K_d Ap	0.191	0.009		AR		0.020	0.004
				LV	0.020		0.404
				LV&CM	0.004	0.404	
K_d B	0.423	0.397					
K_d C	0.007		0.433				
Percolate (potato)	0.003		<0.001	AR		0.010	<0.001
				LV	0.010		0.061
				LV&CM	<0.001	0.061	
Percolate (maize)	0.016		<0.001	AR		0.010	<0.001
				LV	0.010		0.061
				LV&CM	<0.001	0.061	
ET (potato)	0.003		<0.001	AR		0.010	<0.001
				LV	0.010		0.061
				LV&CM	<0.001	0.061	
ET (maize)	0.017		<0.001	AR		0.010	<0.001
				LV	0.010		0.061
				LV&CM	<0.001	0.061	
FC Ap	0.073		<0.001	AR		0.016	<0.001
				LV	0.016		0.051
				LV&CM	<0.001	0.051	
FC B	<0.001		<0.001	AR		0.007	<0.001
				LV	0.007		0.119
				LV&CM	<0.001	0.119	
FC C	0.007		<0.001	AR		0.004	<0.001
				LV	0.004		0.289
				LV&CM	<0.001	0.289	
Ks Ap	0.254		<0.001	AR		<0.001	0.003
				LV	<0.001		1.000
				LV&CM	0.003	1.000	

Ks B	<0.001		<0.001	AR		<0.001	0.001
				LV	<0.001		1.000
				LV&CM	0.001	1.000	
Ks C	<0.001		<0.001	AR		<0.001	0.061
				LV	<0.001		0.061
				LV&CM	0.061	0.061	
Volatilisation (potato)	0.254	0.043		AR		0.032	0.030
				LV	0.032		0.827
				LV&CM	0.030	0.827	
Volatilisation (maize)	0.276	0.041		AR		0.033	0.027
				LV	0.033		0.784
				LV&CM	0.027	0.784	

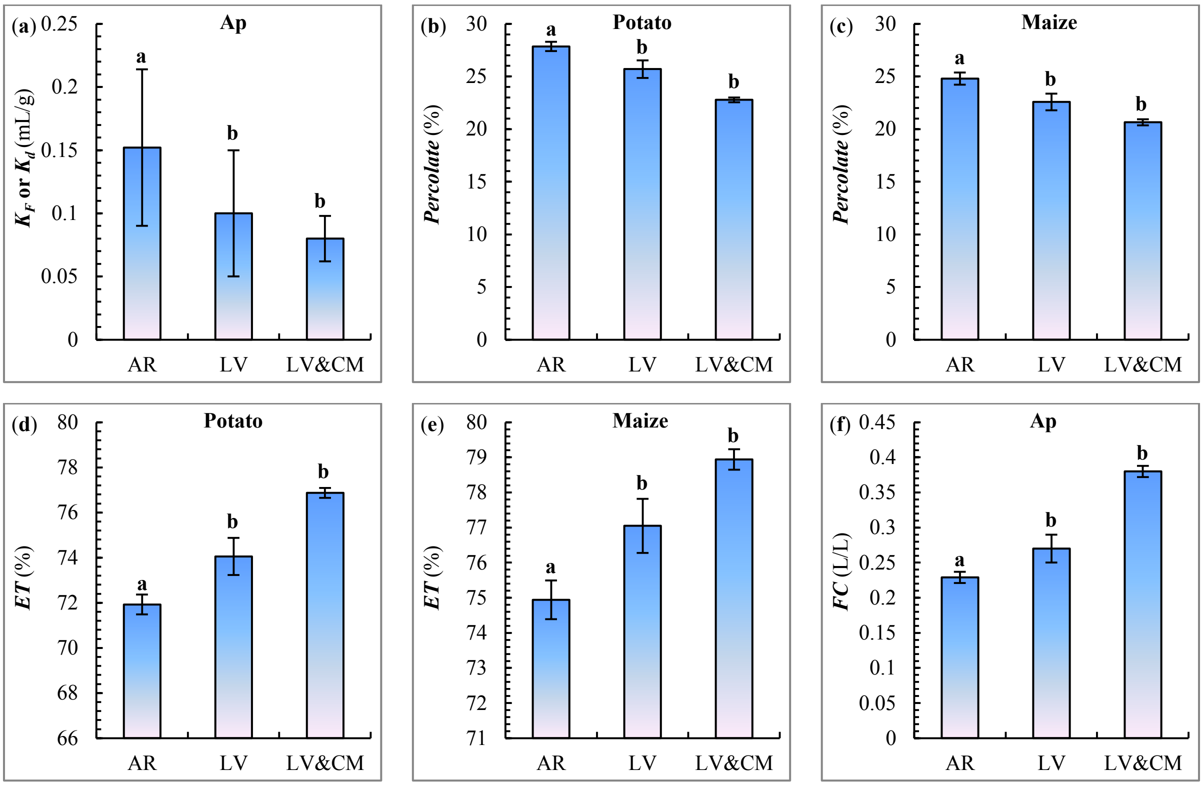

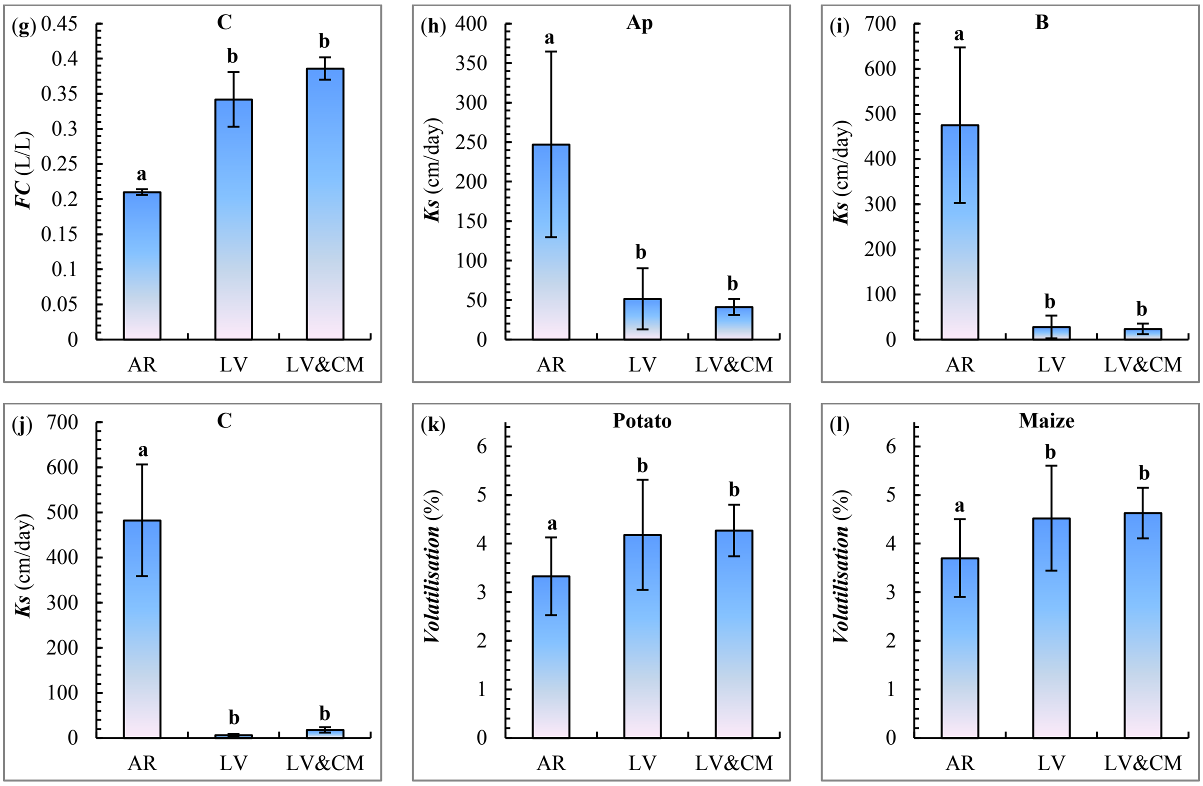

Figure. S7. Comparison of means, standard deviations (error bars), and results of ANOVA analyses (different letters above error bars denote significant differences ($p < 0.05$); for details see Table S4) for the selected soil properties.

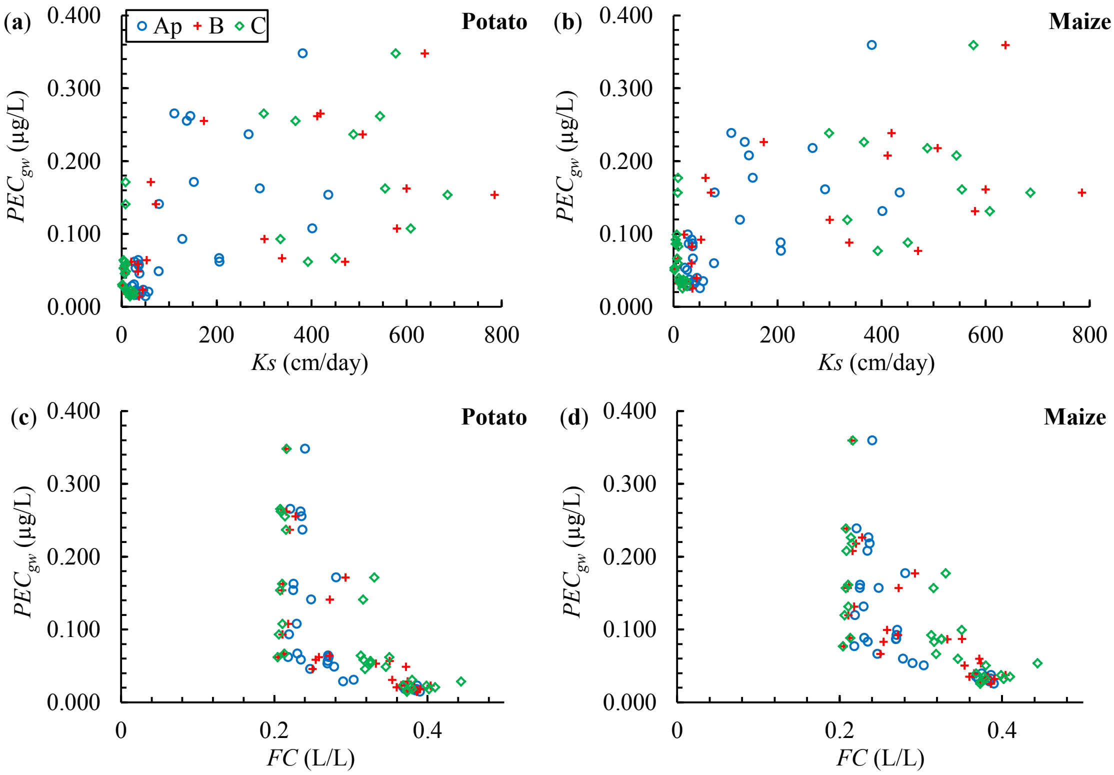

Figure. S8. Relationships between PEC_{gw} and *Ks*, and between PEC_{gw} and *FC* for potato and maize cultivation in the examined soils.

High variance of data in Figure S8a and S8b suggests that this rather nonlinear relationship is weak. However, the Spearman's correlation coefficients between *PECgw* and *Ks Ap, Ks B*, and *Ks C* were significant: 0.673 (p<0.001), 0.741(p<0.001), and 0.449 (p=0.013) for potato, and 0.616 (p<0.001), 0.684 (p<0.001), and 0.389 (p=0.034) for maize cultivation. It is interesting that also the nonlinear relationship between *PECgw* and *FC Ap, FC B*, and *FC C* presented in Figure S8 was significant: -0.792 (p<0.001), -0.823 (p<0.001), and -0.805 (p<0.001) for potato, and -0.750 (p<0.001), -0.783 (p<0.001), and -0.757 (p<0.001) for maize cultivation.

Table S5. Percentiles of *OC* contents in the AR and LV soil groups according to the database and soil collection of the Institute of Agrophysics of the Polish Academy of Sciences in Lublin (Bieganowski et al., 2013).

Percentile	Arenosols			Luvisols		
	Ap	Bw	C	Ap	E	Bt
(n)	(255)	(58)	(51)	(150)	(23)	(13)
99th	3.61	1.72	0.67	2.88	0.78	0.16
95th	2.23	1.09	0.20	1.26	0.65	0.15
90th	1.52	0.68	0.14	1.16	0.57	0.15
85th	1.30	0.51	0.11	1.07	0.44	0.13
80th	1.14	0.36	0.11	0.95	0.39	0.13
75th	1.05	0.32	0.08	0.91	0.37	0.12
70th	0.95	0.30	0.08	0.85	0.36	0.12
65th	0.92	0.23	0.07	0.82	0.35	0.12
60th	0.87	0.21	0.07	0.80	0.33	0.11
55th	0.82	0.20	0.07	0.75	0.29	0.11
50th	0.78	0.18	0.06	0.75	0.28	0.10
45th	0.75	0.17	0.06	0.73	0.26	0.09
40th	0.68	0.16	0.06	0.71	0.26	0.09
35th	0.65	0.14	0.05	0.67	0.24	0.08
30th	0.61	0.12	0.05	0.65	0.20	0.08

25th	0.58	0.11	0.04	0.61	0.19	0.08
20th	0.54	0.10	0.04	0.58	0.16	0.07
15th	0.48	0.09	0.03	0.52	0.15	0.07
10th	0.44	0.08	0.02	0.46	0.13	0.06
5th	0.37	0.05	0.01	0.40	0.12	0.05
1st	0.32	0.04	0	0.32	0.07	0.04

References

Bieganowski, A., et al., 2013. Database of Polish arable mineral soils: a review. Int. Agrophys. 27, 335-350.

FOCUS, FOCUS groundwater scenarios in the EU review of active substances. Report of the FOCUS groundwater scenarios workgroup. Vol. EC Document Reference Sanco/321/2000 rev.2, 202 pp, 2000.

ISO 11277, Soil quality - Determination of particle size distribution in mineral soil material - Method by sieving and sedimentation., 2009.

Klein, M., PELMO (Pesticide Leaching Model)). Version 5.00. User Manual. Fraunhofer Institut for Molecular Biology and Applied Ecology, D-57392 Schmallenberg, Germany. 2018, pp. 30.

Paszko, T., Muszyński, P., 2017. Degradation rates of alachlor, atrazine and bentazone in the profiles of Polish Luvisols. Int. Agrophys. 31, 401-410.
